# Movies and TV Influence Tobacco Use in India: Findings from a National Survey

**DOI:** 10.1371/journal.pone.0011365

**Published:** 2010-06-29

**Authors:** K. Viswanath, Leland K. Ackerson, Glorian Sorensen, Prakash C. Gupta

**Affiliations:** 1 Department of Society, Human Development, and Health, Harvard School of Public Health, Boston, Massachusetts, United States of America; 2 Center for Community-Based Research, Dana-Farber Cancer Institute, Boston, Massachusetts, United States of America; 3 Department of Community Health and Sustainability, University of Massachusetts Lowell, Lowell, Massachusetts, United States of America; 4 Healis-Sekhsaria Institute for Public Health, Navi Mumbai, India; Kenya Medical Research Institute, Kenya

## Abstract

**Background:**

Exposure to mass media may impact the use of tobacco, a major source of illness and death in India. The objective is to test the association of self-reported tobacco smoking and chewing with frequency of use of four types of mass media: newspapers, radio, television, and movies.

**Methodology/Principal Findings:**

We analyzed data from a sex-stratified nationally-representative cross-sectional survey of 123,768 women and 74,068 men in India. All models controlled for wealth, education, caste, occupation, urbanicity, religion, marital status, and age. In fully-adjusted models, monthly cinema attendance is associated with increased smoking among women (relative risk [RR]: 1·55; 95% confidence interval [CI]: 1·04–2·31) and men (RR: 1·17; 95% CI: 1·12–1·23) and increased tobacco chewing among men (RR: 1·15; 95% CI: 1·11–1·20). Daily television and radio use is associated with higher likelihood of tobacco chewing among men and women, while daily newspaper use is related to lower likelihood of tobacco chewing among women.

**Conclusion/Significance:**

In India, exposure to visual mass media may contribute to increased tobacco consumption in men and women, while newspaper use may suppress the use of tobacco chewing in women. Future studies should investigate the role that different types of media content and media play in influencing other health behaviors.

## Introduction

The role of mass media in promoting and reducing tobacco use in the United States is now well-documented [1], [2]. Mass media marketing of tobacco products through direct advertising, as well as through product placement in cultural and entertainment events, has been linked to increased tobacco use [3], [4]. For example, evidence from the United States indicates that higher exposure to smoking in entertainment programming leads to greater initiation among youth possibly through social modeling and by reducing resistance to counter-arguments [1], [2], [5], [6].

At the same time, research has shown that mass media can be successful in discouraging all forms of tobacco use [1]. Exposure to newspaper coverage of tobacco issues has been shown to be related to reduced smoking rates and higher levels of disapproval of smoking behaviors [7]. Anti-tobacco mass media campaigns have also been shown to be effective at reducing smoking rates and increasing the perceived harm from smoking [1], [7]. These campaigns are much stronger when media communications are combined with other strategies of tobacco control, and are dampened by tobacco marketing activities [8].

While much of the published work about tobacco use comes from developed countries, it is now widely agreed that a disproportionate burden resulting from tobacco use is likely to be borne by the developing world. The burden of chronic disease associated with tobacco use is attracting increasing attention in emerging economies such as India where an estimated 700,000–900,000 new cancer cases are diagnosed every year [9] and approximately 250,000 of these cases are directly attributable to the use of tobacco each year [10]. India is the second largest tobacco consumer in the world [11] and tobacco use is the leading cause of cancer of the oral cavity and lung [9] and is a major contributing factor to tuberculosis mortality [12], [13]. Tobacco use is expected to claim nearly 1 million lives in India in 2010 [14], and the total is expected to climb to 1·5 million lives annually by 2020 [15] which will account for 13% of all Indian deaths [16].

Initial research in India has found that specific media content such as media advertising is associated with higher smoking rates [17], and exposure to cigarette brand names or actors smoking on television have been found to be related to increased youth smoking in India [18]. At the same time, anti-smoking messages delivered through the mass media have been shown to reduce smoking in India [18], [19].

Cultural traditions and social norms specific to India play an important role in tobacco use patterns. Contrary to most developed nations, the use of chewing tobacco is widespread in India [20]. According to traditional values in many parts of India, smoking by women is considered taboo; however, the use of smokeless tobacco among these populations is culturally acceptable [10]. The abundance of inexpensive and convenient preparations of smokeless tobacco, coupled with aggressive marketing result in high levels of tobacco chewing, even among women in the country [21]. In addition, the ban on public smoking in India has also led to an increase in the consumption of smokeless tobacco, and the tobacco industry has started focusing more on advertising smokeless tobacco products which are not affected by current tobacco control policies [22].

This paper focuses on understanding the extent to which mass media use is related to tobacco use in India given that media use may expose the audience to both pro and anti-tobacco content. The assumption is that understanding and documenting patterns of the differential effects of media use on tobacco use may help develop strategic communication campaigns to stem tobacco use. In line with this assumption, this paper focused on one overriding research question: to what extent are access to and use of mass media related to tobacco use among men and women in India after controlling for socioeconomic and demographic characteristics. In addition to assessing the unique patterns of media use and smoked and chewed tobacco among men and women in India, this assessment can serve as a model for similar investigations in other developing countries.

## Methods

### Data Source

This project used the 2005–2006 National Family Health Survey, the Indian version of the Demographic and Health Surveys which are administered by ICF Macro in 75 countries [23]. This survey is a nationally-representative cross-sectional study designed to provide information about adult AIDS attitudes and behaviors and maternal and child health issues.

### Sampling Plan, Study Population and Sample Size

A multistage sampling procedure began by stratifying all 29 states into urban and rural areas. The sample size for each state was selected proportional to the size of the state's urban and rural populations. Primary sampling units were defined as census enumeration blocks in urban areas and as villages in rural areas and were selected within each state according to probability proportional to size. Households were selected at random from within each primary sampling unit.

Face-to-face interviews were conducted with an adult member of 109,041 selected households to obtain demographic information about the households and family members, with a household response rate of 97·7% [23]. This household survey identified 131,596 female residents aged 15 to 49 eligible to participate in a survey of maternal and child health which also collected information about mass media use and tobacco use. A total of 124,385 women participated in this survey for an individual response rate of 94·5%. Of these women, 617 were missing information leaving 123,768 women in the analytic sample. In a random sample of selected households, 85,373 men aged 15–54 were invited to answer questions about mass media and tobacco use. A total of 74,369 men responded to this survey for a response rate of 87·1% [23]. Of these men, 301 were missing information leaving 74,068 men in the analytic sample.

### Outcome Measures

#### Tobacco use

Individuals were asked “Do you currently smoke cigarettes or bidis?” with a bidi being a thin, hand-rolled cigarette traditionally smoked in India. A subsequent question asked “In what other form do you currently smoke or use tobacco?” with possible answers including cigar/pipe, paan masala, ghutka, and other chewing tobacco. Paan masala and ghutka are mixtures of chewing tobacco, areca nut, and slaked lime that typically contain other flavorings as well. Those who replied that they smoked cigarettes, bidis, cigars, or pipes were considered to be smokers. Those who identified themselves as using paan masala, ghutka, or other chewing tobacco were considered to be tobacco chewers.

#### Exposure

Individuals were asked how often they read newspapers, listen to the radio, and watch television with possible answers being “almost every day” [daily], “at least once a week” [weekly], “less than once a week” [occasionally], and “not at all” [never]. An additional question asked, “Do you usually go to a cinema hall or theatre to see a movie at least once a month?” with a binary response of yes or no.

#### Covariates

Wealth, defined in terms of living environment and material possessions, has been documented as a valid measure of socioeconomic status in the context of India [24]. Following an established methodology that is consistent with other research on India, each individual was assigned a wealth score created by weighting responses regarding household possessions and characteristics with a factor analysis procedure and dividing the results into quintiles [23], [25]. Education was defined according to important milestones in the Indian educational system: 0 years, 1–5 years, 6–10 years, 11–12 years, and 13 or more years. Individuals were categorized as to whether they belonged to one of three legislatively-defined socially marginalized groups: scheduled castes, scheduled tribes, and “other backward classes.” Scheduled castes and scheduled tribes are the groups that have experienced the greatest burden of deprivation within the Indian social hierarchy while other backward classes have suffered less severe deprivation [26]. Those who did not identify as any of these three marginalized classes were considered to be members of the general class. Occupation was created from self-reported jobs and categorized as not working, performing non-manual work, performing agricultural work, or performing non-agricultural manual work. We used 2001 Indian National Census figures to define each primary sampling unit as within an urban or rural area. Religion was categorized from household reports as Hindu, Muslim, Christian, or other. For marital status, those who were divorced, widowed, separated, or never married were grouped as “unmarried.” Age was categorized in five year increments.

#### Statistical analysis

Due to social patterning of tobacco use in India, we elected a priori to stratify all analyses by sex. For each tobacco use outcome for each sex, we created one model that included all three categorical mass media use variables measuring television, radio, and newspaper use, and the binary variable measuring monthly movie watching. Each model was fully adjusted for all socioeconomic and demographic covariates including wealth, education, caste, occupation, urbanicity, religion, marital status, and age. Because outcomes in the analyses were not rare, particularly for men's tobacco use, odds ratios could not provide an accurate assessment of risk. In place of this, we used a generalized estimating equation modified Poisson regression approach with robust error variance to produce direct assessments of the relative risks [27]. All models accounted for survey weights and clustering within primary sampling units, and states were included as dummy variables.

#### Ethics statement

The data collection was administered by the International Institute for Population Sciences (IIPS) in Mumbai, India under the direction of the Ministry of Family Health and Welfare of the Government of India. Before participating in the household survey and individual survey, all participants were asked to provide informed consent after being read a document emphasizing the voluntary nature of this project, outlining potential risks, and explaining that the information gathered would be used to assess health needs and better plan health services. Informed consent was obtained verbally from all participants due to the fact that a substantial proportion of the participants in this study were illiterate. These data-collection procedures were specifically reviewed and approved by an independent ethics review conducted by IIPS. Approval for the use of this data for the specific purpose of this study was granted by the Institutional Review Boards at ICF Macro, the Harvard School of Public Health, and the University of Massachusetts Lowell.

## Results

### Descriptive characteristics

Among women, 1·5% smoked and 8·4% chewed tobacco (Table 1). At least occasional newspaper, radio, and television use was reported among 36·3%, 44·3%, and 65·6% of the women in the population, respectively. Smoking and chewing were reported by 33·6% and 36·4% of men, respectively (Table 2). Of men in the population, 68·3% used newspapers, 69·5% used radio, and 82·3% used television at least occasionally. Indicators of socioeconomic position, wealth and education are strongly and inversely associated with smoking and chewing (Tables 1 and 2). Although each type of media use was more common in an urban rather than rural context, there was sufficient heterogeneity in media use by rural/urban location to provide an adequate sample size in each cell to permit the use of multivariable regressions without stratifying the samples (Table 3).

**Table 1:** Socioeconomic, demographic, and media use characteristics by tobacco use among women in the 2005–2006 National Family Health Survey of India.

	Full Sample		Smokers		Tobacco Chewers	
	N	Weighted %^1^	N	Weighted %^2^	N	Weighted %^3^
Total	123768	100·0	1929	1·5	13236	8·4
Newspaper use						
Never	67423	63·7	1531	2·3	8927	11·2
Occasionally	18894	13·4	154	0·2	1625	4·6
Weekly	16395	10·4	132	0·1	1393	3·4
Daily	21056	12·5	112	0·1	1291	2·1
Radio use						
Never	65075	55·7	1153	1·9	6807	
Occasionally	19322	15·5	290	1·2	2342	9·0
Weekly	15204	11·6	213	1·1	1623	7·7
Daily	24167	17·1	273	0·8	2464	6·6
Television use						
Never	30910	34·4	894	3·0	4248	11·7
Occasionally	12708	10·5	295	1·7	1981	11·4
Weekly	15016	11·4	278	1·2	2095	9·2
Daily	65134	43·7	462	0·3	4912	4·8
Movie use						
Less than once a month	115679	94·4	1847	1·5	12639	8·6
At least once a month	8089	5·6	82	0·7	597	4·1
Wealth						
1st (lowest) quintile	14031	17·5	472	3·6	2750	16·8
2nd quintile	17549	19·0	467	2·3	2603	11·2
3rd quintile	23532	20·2	450	1·3	3069	7·9
4th quintile	29980	21·0	306	0·6	2946	5·5
5th (highest) quintile	38676	22·4	234	0·2	1868	2·6
Education (years)						
None	39747	40·7	1336	3·3	6223	13·5
1 to 5	17481	14·5	261	0·7	2397	9·8
6 to 10	42208	30·2	260	0·1	3521	4·2
11 to 12	11423	7·2	30	0·03	577	1·5
13 or more	12909	7·3	42	0·1	518	1·1
Caste						
Scheduled caste	20530	18·8	389	2·4	2137	10·6
Scheduled tribe	16431	8·1	720	2·6	4519	21·2
Other backward class	39470	39·6	444	1·4	2807	5·9
General class	47337	33·5	376	0·8	3773	7·0
Occupation						
None	73946	57·3	754	0·9	5812	6·0
Non-manual	9764	5·1	135	0·7	1293	5·9
Agricultural	23911	25·2	769	2·9	3669	12·0
Manual	16147	12·4	271	1·6	2462	13·3
Urbanicity						
Rural	67069	67·2	1448	2·0	8540	9·8
Urban	56699	32·8	481	0·5	4696	5·5
Religion						
Hindu	89564	80·5	1178	1·5	8172	8·3
Muslim	16621	13·6	216	1·7	1467	9·1
Christian	10909	2·5	407	1·2	2902	10·4
Other	6674	3·4	128	0·4	695	6·9
Marital status						
Married	87500	74·8	1603	1·8	10274	9·5
Unmarried	36268	25·2	326	0·6	2962	5·0
Age (years)						
15 to 19	23844	19·9	61	0·1	1096	3·0
20 to 24	22699	18·3	152	0·4	1700	4·6
25 to 29	20542	16·4	199	1·0	2156	7·5
30 to 34	17788	14·2	291	1·7	2324	10·4
35 to 39	16057	12·7	405	2·7	2380	12·9
40 to 44	13084	10·5	423	3·2	2007	13·8
45 to 49	9754	7·9	398	4·0	1573	14·7

1 Indicates the percentage of women in the population with that particular characteristic.

2 Indicates the percentage of women with that particular socioeconomic, demographic, or media use characteristic who smoke.

3 Indicates the percentage of women with that particular socioeconomic, demographic, or media use characteristic who chew tobacco.

**Table 2:** Socioeconomic, demographic, and media use characteristics by tobacco use among men in the 2005–2006 National Family Health Survey of India.

	Full Sample		Smokers		Tobacco Chewers	
	N	Weighted %^1^	N	Weighted %^2^	N	Weighted %^3^
Total	74068	100·0	24285	33·6	25511	36·4
Newspaper use						
Never	19954	31·7	8772	45·0	8313	43·6
Occasionally	10900	15·5	3679	32·8	4272	41·2
Weekly	13736	18·3	4197	29·7	4872	36·0
Daily	29478	34·5	7637	25·6	8054	27·8
Radio use						
Never	21682	30·5	7075	34·5	6690	32·5
Occasionally	17630	25·5	6125	35·2	6727	41·4
Weekly	14391	19·6	4856	34·1	5377	39·8
Daily	20365	24·4	6229	30·4	6717	33·2
Television use						
Never	9178	17·7	3803	41·3	3713	43·5
Occasionally	11739	19·6	4715	39·7	5347	46·4
Weekly	11947	16·3	4276	35·5	4730	39·4
Daily	41204	46·3	11491	27·4	11721	28·3
Movie use						
Less than once a month	58760	80·5	19958	34·7	21126	37·6
At least once a month	15308	18·5	4327	28·8	4385	31·0
Wealth						
1st (lowest) quintile	7053	15·8	3092	44·0	3519	50·9
2nd quintile	10230	18·1	4224	40·7	4460	44·9
3rd quintile	14792	20·4	5490	36·3	5666	37·0
4th quintile	19272	22·1	6121	29·9	6568	32·1
5th (highest) quintile	22721	23·6	5358	22·4	5298	23·6
Education (years)						
None	10815	18·8	5501	51·3	4606	45·1
1 to 5	11241	16·7	4861	44·0	4758	43·7
6 to 10	31081	40·4	9501	29·5	11017	36·7
11 to 12	9537	11·6	2004	19·2	2528	25·3
13 or more	11394	12·4	2418	19·9	2602	22·8
Caste						
Scheduled caste	12626	18·8	4699	40·0	4610	39·4
Scheduled tribe	8930	8·2	3729	38·0	4281	50·1
Other backward class	26682	39·1	8222	32·2	8536	35·6
General class	25830	34·0	7635	30·7	8084	32·3
Occupation						
None	10922	12·7	1418	10·2	1820	14·4
Non-manual	18798	21·4	5419	29·5	5754	31·3
Agricultural	17251	29·8	6843	39·7	7153	41·6
Manual	27097	36·0	10605	39·4	10784	42·8
Urbanicity						
Rural	36009	63·5	12992	36·1	13715	39·5
Urban	38059	36·5	11293	29·3	11796	31·0
Religion						
Hindu	54499	82·0	17395	33·8	18380	37·1
Muslim	9548	12·4	3287	36·9	3169	35·8
Christian	6627	2·3	2727	33·9	2820	28·0
Other	3394	3·3	876	16·3	1142	25·6
Marital status						
Married	44711	64·4	17818	41·0	17377	40·9
Unmarried	29357	35·6	6467	20·2	8134	28·1
Age (years)						
15 to 19	13009	17·5	1690	12·2	2649	22·0
20 to 24	12422	16·1	3411	26·8	4423	38·6
25 to 29	11022	14·6	3869	35·0	4446	42·2
30 to 34	9725	13·1	3559	37·6	3940	42·3
35 to 39	9104	12·5	3658	42·3	3562	40·6
40 to 44	7757	10·9	3374	45·2	2786	38·0
45 to 49	6514	9·1	2807	45·1	2282	36·1
50 to 54	4515	6·2	1917	45·4	1423	33·9

1 Indicates the percentage of men in the population with that particular characteristic.

2 Indicates the percentage of men with that particular socioeconomic, demographic, or media use characteristic who smoke.

3 Indicates the percentage of men with that particular socioeconomic, demographic, or media use characteristic who chew tobacco.

**Table 3:** Media use characteristics by rural/urban strata among women and men in India.

	Women				Men			
	Urban		Rural		Urban		Rural	
	N	Weighted %	N	Weighted %	N	Weighted %	N	Weighted %
Total	56699	100.0	67069	100.0	38059	100.0	36009	100.0
Newspaper use								
Never	21842	42.6	45581	74.0	6781	18.3	13173	39.4
Occasionally	9264	15.7	9630	12.3	4554	12.1	6346	17.5
Weekly	9711	15.7	6684	7.8	6655	17.3	7081	18.8
Daily	15882	25.9	5174	6.0	20069	52.3	9409	24.3
Radio use								
Never	28960	53.8	36115	56.7	11558	31.5	10124	30.0
Occasionally	8458	14.5	10864	16.0	8592	23.4	9038	26.7
Weekly	6896	11.3	8308	11.8	7236	18.8	7155	20.0
Daily	12385	20.4	11782	15.5	10673	26.3	9692	23.3
Television use								
Never	5094	11.0	25816	45.8	1871	6.1	7307	24.4
Occasionally	3883	7.0	8825	12.3	3497	10.2	8242	25.0
Weekly	6127	10.2	8889	11.9	5064	13.4	6883	18.0
Daily	41595	71.8	23539	30.0	27627	70.3	13577	32.6
Movie use								
Less than once a month	51297	90.8	64382	96.1	28235	75.0	30525	85.2
At least once a month	5402	9.2	2687	3.9	9824	25.0	5484	14.8

### Media and smoking

Smoking was more common among women (relative risk [RR]: 1·55; 95% confidence interval [CI]: 1·04–2·31) who attended the cinema monthly compared to those who did not (Table 4). While occasional newspaper use was associated with lower smoking prevalence among women (RR: 0·72; 95% CI: 0·57–0·89), no relation was found between daily newspaper use and smoking. No other associations were found between media use and smoking among women. Among men, no association was found of newspaper use or radio use with smoking. Smoking was more common among men who watched television daily compared to those who never watched television (RR: 1·06; 95% CI: 1·02–1·11) and among men (RR: 1·17; 95% CI: 1·12–1·23) who attended the cinema monthly compared to those who did not.

**Table 4:** Adjusted relative risk (RR) and 95% confidence intervals (CI) for the association between media use and tobacco use among men and women in the 2005–2006 National Family Health Survey.

	Women Smoking		Men Smoking		Women Tobacco Chewing		Men Tobacco Chewing	
	RR	95% CI	RR	95% CI	RR	95% CI	RR	95% CI
Newspaper use								
Never (reference)	1		1		1		1	
Occasionally	0·62	0·40–0·98	1·02	0·97–1·07	0·97	0·88–1.08	1·00	0·96–1·05
Weekly	0·73	0·38–1·39	1·03	0·97–1·08	0·90	0·79–1·02	0·98	0·94–1·03
Daily	0·80	0·44–1·46	1·00	0·95–1·06	0·82	0·70–0·96	0·98	0·93–1·03
Radio use								
Never (reference)	1		1		1		1	
Occasionally	0·72	0·57–0·89	1·02	0·98–1·06	1·06	0·99–1·14	1·06	1·02–1·10
Weekly	0·76	0·58–0·98	1·05	1·00–1·09	1·07	0·98–1·17	1·06	1·02–1·11
Daily	1·11	0·87–1·41	1·03	0·99–1·08	1·15	1·05–1·26	1.02	0·98–1·06
Television use								
Never (reference)	1		1		1		1	
Occasionally	1·19	0·95–1·48	1·06	1·02–1·12	1·09	1·00–1·18	1·09	1·04–1·14
Weekly	1·09	0·85–1·38	1·11	1·06–1·16	1·16	1·07–1·26	1·11	1·05–1.16
Daily	0·89	0·68–1·16	1·11	1·06–1·17	1·16	1·07–1·27	1·12	1·07–1·18
Movie use								
Less than once per month (reference)	1		1		1		1	
At least once a month	1·55	1·04–2·31	1·17	1·12–1·23	1·04	0·90–1·21	1·15	1·11–1·20

Note: Results are mutually adjusted for the other media use variables as well as wealth, education, caste, occupation, location, religion, age, marital status, and state.

### Media and tobacco chewing

While daily newspaper use was associated with lower likelihood of tobacco chewing among women (RR: 0·82; 95% CI: 0·70–0·96), those women who watched television (RR: 1·16; 95% CI: 1·07–1·27) and listened to the radio (RR: 1·15; 95% CI: 1·06–1·26) every day had higher likelihood of tobacco chewing (Table 4). There was no association between watching movies and tobacco chewing among women.

Among men, newspaper and radio use were not associated with tobacco chewing (Table 4). Men who watched television daily (RR: 1·12; 95% CI: 1·07–1·18) and watched a movie at least once a month (RR: 1·15; 95% CI: 1·11–1·20) were more likely to chew than those who did not use these media.

## Discussion

We found several distinct patterns in our investigation into mass media use and tobacco use among Indian adults. Exposure to television and monthly attendance at the cinema was associated with higher likelihood of smoking among men, while monthly attendance at the cinema was associated with higher likelihood of smoking among women. Use of television and monthly attendance at the cinema was also associated with increased tobacco chewing among both men and women. These findings are consistent with previous research from the United States [1] and India [17], [18]. Newspaper use, however, was associated with decreased tobacco chewing among women only. To our knowledge, this represents the first nationally-representative study finding a relationship between media and tobacco use among Indian adults.

The literature on media effects posits that media exposure may influence behaviors in two ways: frequency of exposure to different media and the content in the media [28]. The data in this study clearly demonstrate that use of media is independently associated with tobacco use. More importantly, the differential associations of media types on tobacco use suggest that the content in the media types vary and that this content likely accounts for the differences in the associations. Since this dataset contains no information about media content, however, we can only speculate about this in our paper. Different media genres are likely to play different roles in tobacco use. Advertising and entertainment media are more likely to be receptive to pro-tobacco content given the heavy promotion of tobacco use in advertising and incidence of smoking in movies. The tobacco industry spends billions of dollars on tobacco promotion as has been widely documented [1]. Moreover entertainment media, particularly movies, are known to carry incidents of smoking by the characters [1]. Both of these, in the absence of counter-arguments, could lead to pro-tobacco beliefs and thus promote tobacco use. It is likely that Indian visual media are more hospitable to pro-tobacco messages compared to other media. Newspapers on the other hand do carry tobacco advertising but also are likely to carry stories on harmful effects of smoking [1]. As a result, it is likely that the impact of newspapers on pro-tobacco beliefs and behaviors may be more muted.

Lastly the nature of the audience also matters. Newspaper readers are more likely to be from higher SES, a group that is less likely to use tobacco compared to the audience for visual media. It is possible that newspapers readers could be more critical consumers of media content.

Another issue worth speculating about is the literacy levels of the participants. While we do not have direct measures of literacy, it is highly likely that education or formal schooling is related to differential media use which in turn may influence the effects of mass media.

Our results are subject to the same caveats as are found with any cross-sectional study. Reverse causation is a possibility, although it is unlikely that tobacco use would cause individuals to increase their use of mass media. It is also important to note that while our data were limited to four traditional media types, other mass media channels such as billboards, cell phones, the Internet, and promotional items could also be important methods of communicating tobacco-related messages. An additional concern for this observational study is uncontrolled confounding. We did, however, adjust all models for a number of social and demographic variables including four measures of socioeconomic status. Finally, this study does not provide any specificity on the nature of the media exposure, including what type of content consumers of each media type were actually exposed to. Future studies should assess the nature of the content of each media type to determine what kind of messages may be promoting or suppressing tobacco use behaviors.

The study has several important strengths as well. These include the large sample that is representative of the entire Indian population. In addition, India is a key developing country that can provide insight for assessing other developing nations with strong mass media traditions.

This study identified associations of visual, audio, and print mass media use with tobacco chewing and smoking in a nationally-representative sample of Indian adults. These findings provide evidence that exposure to pro-tobacco content in television and cinema may promote tobacco use among men and women in India. This suggests clear directions for actions to curb pro-tobacco messages in these media could serve to reduce the use of tobacco and subsequent tobacco-related illnesses in India. Future studies should examine tobacco-related media content in visual, audio, and print media to obtain a more complete picture of information environment about tobacco use which could serve to help develop appropriate health promotion interventions. This information could assist medical, public health, and public policy professionals in designing programs to reverse the recent increase in tobacco use and promote cessation among individuals in India.
